# Salting-Out of DNA Origami Nanostructures by Ammonium Sulfate

**DOI:** 10.3390/ijms23052817

**Published:** 2022-03-04

**Authors:** Marcel Hanke, Niklas Hansen, Ruiping Chen, Guido Grundmeier, Karim Fahmy, Adrian Keller

**Affiliations:** 1 Technical and Macromolecular Chemistry, Paderborn University, Warburger Str. 100, 33098 Paderborn, Germany; marcelha@mail.uni-paderborn.de (M.H.); niklashansen94@aol.de (N.H.); ruiping@campus.uni-paderborn.de (R.C.); g.grundmeier@tc.uni-paderborn.de (G.G.); 2 Helmholtz-Zentrum Dresden-Rossendorf, Institute of Resource Ecology, Bautzner Landstrasse 400, 01328 Dresden, Germany; k.fahmy@hzdr.de; 3 Center for Molecular and Cellular Bioengineering, Technische Universität Dresden, 01062 Dresden, Germany

**Keywords:** DNA origami, DNA nanotechnology, ammonium sulfate, precipitation, salting-out

## Abstract

DNA origami technology enables the folding of DNA strands into complex nanoscale shapes whose properties and interactions with molecular species often deviate significantly from that of genomic DNA. Here, we investigate the salting-out of different DNA origami shapes by the kosmotropic salt ammonium sulfate that is routinely employed in protein precipitation. We find that centrifugation in the presence of 3 M ammonium sulfate results in notable precipitation of DNA origami nanostructures but not of double-stranded genomic DNA. The precipitated DNA origami nanostructures can be resuspended in ammonium sulfate-free buffer without apparent formation of aggregates or loss of structural integrity. Even though quasi-1D six-helix bundle DNA origami are slightly less susceptible toward salting-out than more compact DNA origami triangles and 24-helix bundles, precipitation and recovery yields appear to be mostly independent of DNA origami shape and superstructure. Exploiting the specificity of ammonium sulfate salting-out for DNA origami nanostructures, we further apply this method to separate DNA origami triangles from genomic DNA fragments in a complex mixture. Our results thus demonstrate the possibility of concentrating and purifying DNA origami nanostructures by ammonium sulfate-induced salting-out.

## 1. Introduction

DNA origami nanostructures, first introduced in 2006 [1], have developed into widely used, highly versatile tools for addressing important problems and challenges in biophysics [2,3], biomedicine [4,5], molecular [6,7], structural [8,9], and chemical biology [10,11], sensing [12,13], microscopy [14,15], and many other fields of fundamental and applied research. Although these applications have benefited from the numerous advantages that make DNA origami nanostructures superior to other, more conventional nanostructures, such as high biocompatibility [16,17], high stability in comparatively harsh environments [18,19], and the unprecedented possibility to arrange molecular species with sub-nanometer accuracy [11,20], there are still several challenges that need to be overcome for this technology to enter real-world applications. This in particular concerns the amounts and concentrations of the assembled DNA origami nanostructures that reasonably can be obtained. Since DNA origami assembly relies on the folding of a single-stranded scaffold upon hybridization with multiple oligonucleotide staples at a large molar excess, the total amount of assembled DNA origami nanostructures at a given assembly yield directly correlates with the amount of available scaffold. The achievable concentration, on the other hand, is mostly determined by the available scaffold and staple concentrations and the molar excess of each of the around 200 staples over the scaffold. Consequently, numerous previous studies have examined different techniques with regard to their potential to efficiently purify and also to concentrate DNA origami nanostructures at high yield [21,22,23,24,25,26]. All of these techniques have their own advantages and disadvantages, depending on the actual task and application. For instance, the widely employed spin filtering method is fast and straightforward for purifying assembled DNA origami nanostructures from excess staples. However, the required spin filters are available only with a rather narrow selection of molecular weight cut-offs, which poses a severe limitation when one attempts to separate different high-molecular weight species. This, in particular, concerns hierarchical DNA origami assemblies that have numerous applications in nanoelectronics [27,28] and biomedicine [29,30] but often require efficient purification from excess DNA origami monomers [24]. For such applications, gel electrophoresis is better suited [26]. However, this technique is rather labor-intensive, time-consuming, and exposes the DNA nanostructures to intercalating dyes, which alter their structural and mechanical properties [31,32] and may subsequently be released into the physiological environment [33]. To overcome these issues, sequential pull-down purification of hierarchical DNA origami assemblies using magnetic beads was recently demonstrated, which, however, is again rather time-consuming and labor-intensive, and requires specific DNA origami modifications that enable the selective binding of one species over the other [24].

Another common method for DNA origami purification is PEG precipitation [23,25,33,34,35,36,37,38,39,40]. Here, the assembled DNA origami nanostructures are precipitated as a condensed pellet that, after removal of the supernatant, can be redissolved in a smaller volume to achieve higher concentrations. Precipitation is a result of steric exclusion of the bulky DNA origami nanostructures by the long PEG polymers, which are then concentrated in the extra-polymeric space until their solubility limit is exceeded [41]. This mechanism is a subclass of salting-out [42] and relies mostly on non-specific interactions between the involved polymer species. Therefore, PEG precipitation is a universal and long-established method for the purification of various biopolymers, i.e., double-stranded (ds) DNA fragments [43], plasmids [44], and also proteins [45].

A conceptually similar method routinely used for protein purification and concentration is ammonium sulfate precipitation [46,47]. In this classical salting-out, the added salt ions will neutralize charges at the protein surface and simultaneously compete with the protein surface for water molecules, so that at high salt concentrations, protein structure is stabilized not by protein–water but by protein–protein interactions, which leads to protein precipitation [48]. Ammonium sulfate is particularly effective in this regard because both of its ions, NH_4_^+^ and SO_4_^2−^, are located at the kosmotropic ends of the respective Hofmeister series [46,47]. A general advantage of ammonium sulfate-induced protein precipitation is that the (NH_4_)_2_SO_4_ concentration at which precipitation occurs depends strongly on the molecular weight of the protein, so that it can be used for separating different protein species from complex mixtures such as lysates [47].

In the context of DNA purification, ammonium sulfate precipitation is typically used only to remove the proteinaceous components of cell extracts, with the DNA fraction remaining suspended in solution [49]. However, DNA origami nanostructures are, in some aspects, closer to folded proteins than to genomic dsDNA. Their assembly for instance shares some similarities with the folding of proteins [50,51], and their bulky 3D structure has to be stabilized by the specific or non-specific binding of counterions [52,53]. Furthermore, DNA origami nanostructures were found to be similarly susceptible to chaotropic salt denaturation as proteins [18,54]. Therefore, we speculated that they might also undergo salting-out in concentrated ammonium sulfate solutions and that this might enable the straightforward separation of different DNA species. To test this hypothesis, we centrifuged solutions of different DNA origami shapes in the presence of 3 M (NH_4_)_2_SO_4_ and characterized their segregation by UV absorption and atomic force microscopy (AFM). We found that after centrifugation all DNA origami shapes were concentrated in the bottom 20% of the sample volume. After removal of the top 80% of the solution, the concentrated DNA origami could be resuspended in a (NH_4_)_2_SO_4_-free buffer without apparent formation of aggregates or loss of structural integrity. Our results thus demonstrate the possibility of concentrating DNA origami nanostructures by ammonium sulfate-induced salting-out. This appears to be facilitated largely by the compact structure of the DNA origami nanostructures compared to dsDNA, which enabled us to separate DNA origami nanostructures from a complex mixture containing both DNA origami and genomic dsDNA.

## 2. Results

**Figure 1:**
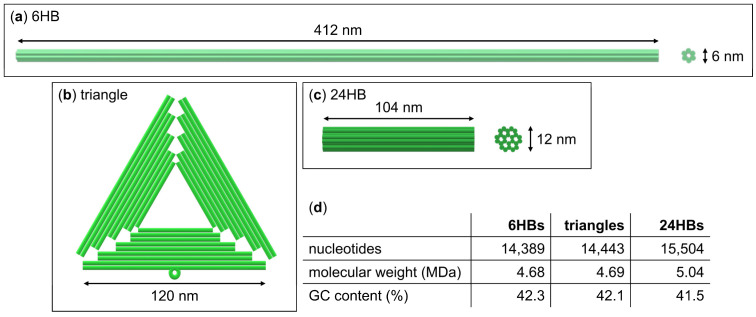
Schematic representations and properties of the DNA origami nanostructures employed in the present work. (**a**) 6HB with nominal dimensions; (**b**) Triangle with nominal dimensions; (**c**) 24HB with nominal dimensions; (**d**) Comparison of total number of nucleotides, molecular weight, and GC content of the three DNA origami designs.

**Figure 2:**
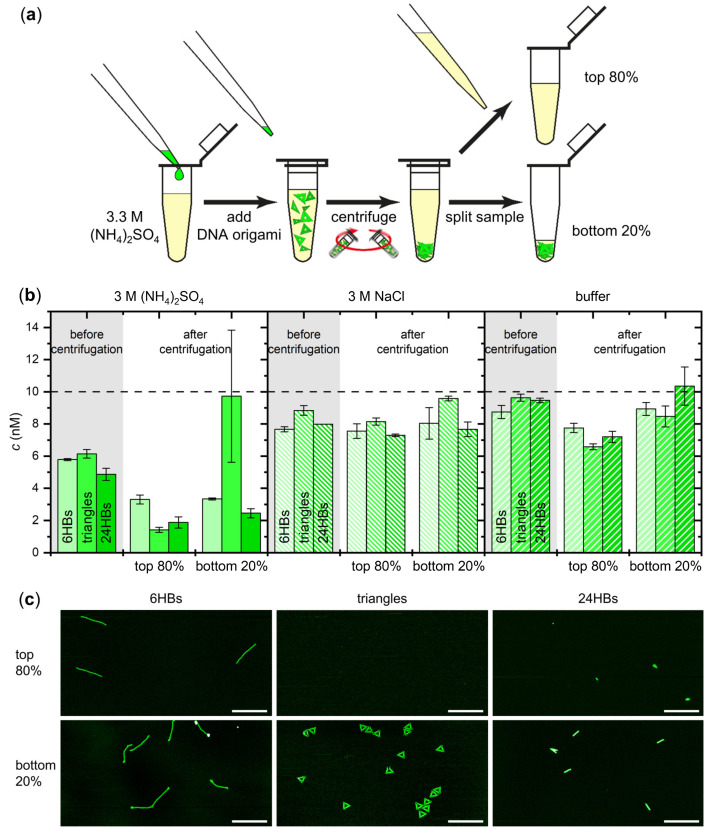
Salting-out of different DNA origami shapes in 3 M (NH_4_)_2_SO_4_. (**a**) Workflow of DNA origami salting-out. DNA origami nanostructures are added to 3.3 M (NH_4_)_2_SO_4_ in assembly buffer, resulting in a final (NH_4_)_2_SO_4_ concentration of 3 M. After centrifugation, the sample is split into two fractions, i.e., the top 80% and the bottom 20% of the total sample volume. Both fractions are then further analyzed using UV absorption and AFM; (**b**) Concentrations of the DNA origami 6HBs, triangles, and 24HBs before and after centrifugation determined by UV absorption. The plots compare the concentrations obtained in the presence of 3 M (NH_4_)_2_SO_4_ (left), 3 M NaCl (center), and in assembly buffer without additional salt (right), respectively. The horizontal broken line indicates the nominal starting concentration; (**c**) Representative AFM images of the different DNA origami nanostructures in the top 80% and the bottom 20% fractions after centrifugation in 3 M (NH_4_)_2_SO_4_. Scale bars are 500 nm and height scales are 4 nm (6HBs), 3 nm (triangles), and 7 nm (24HBs), respectively.

As a first step, we set out to establish whether DNA origami nanostructures are susceptible toward (NH_4_)_2_SO_4_ salting-out at all. For this, we selected three rather different DNA origami shapes, i.e., a quasi-1D six-helix bundle (6HB) [55], a 2D triangle [1], and a 3D 24-helix bundle (24HB) (see Figure 1) [33]. While these three DNA origami nanostructures have similar molecular weights and GC contents around 5 MDa and 42%, respectively, their shapes are very different, ranging from the filament-like 6HB to the rather compact, almost particle-like 24HB. Purified samples of each of the selected DNA origami nanostructures were mixed with assembly buffer (10 mM Tris-acetate at pH 8.0 with 10 mM MgAc_2_) with additional 3.3 M (NH_4_)_2_SO_4_ (see Figure 2a). This resulted in concentrations of 10 nM DNA origami and 3 M (NH_4_)_2_SO_4_ in the final sample. Because the solubility limit of (NH_4_)_2_SO_4_ is only about 4 M [47], we could not achieve any higher (NH_4_)_2_SO_4_ concentrations. Interestingly, mixing the DNA origami with the (NH_4_)_2_SO_4_ solution resulted in an immediate reduction of the DNA origami concentration for all three DNA origami shapes as determined by UV absorption (see Figure 2b), which already hints at a possible salting-out. The sample solutions were then centrifuged for 90 min at 14,000 rcf at 18 °C, after which each sample was split into two fractions, i.e., the top 80% and the bottom 20% of the total sample volume (see Figure 2a). After gentle mixing, the DNA origami concentration in each fraction was measured by UV absorption.

As can be seen in Figure 2b for all three DNA origami shapes, the top 80% of the sample volume has a drastically reduced DNA origami concentration, both with respect to the nominal concentration and the already reduced concentration before centrifugation. In the bottom 20%, similar concentrations as in the top 80% are observed for the helix bundles, whereas the DNA origami triangles show a concentration close to the nominal value of the initial concentration of 10 nM. However, the DNA origami triangles also show a rather large sample-to-sample spread in the concentration of the bottom 20% (see error bars in Figure 2b). This large spread indicates that the gentle mixing of these fractions before the concentration measurements did not result in homogeneous samples, which is indicative of the formation of solid or semi-solid precipitates with low solubility. In line with this interpretation, for all three DNA origami shapes, the concentrations determined after centrifugation indicate a loss of DNA material, which also points toward a precipitation of the DNA origami nanostructures at the bottom of the tubes. Since the concentration measurements were conducted with 1 µL aliquots (see Section 4.3) taken from the complete sample volume, the resulting snapshots do not accurately reflect the overall composition of such heterogeneous samples. Therefore, we believe that the observed variations between the different DNA origami samples originate mostly in the formation of more or less insoluble precipitates at the bottom of the tubes. In pure assembly buffer as well as in 3 M NaCl, however, higher and more similar concentrations close to the nominal initial concentration were observed for all three DNA origami shapes before and after centrifugation (see Figure 2b), which is a clear indication that DNA origami nanostructures are susceptible to ammonium sulfate-induced salting-out.

The concentration measurements in Figure 2b were complemented with molecular-level data to assess possible DNA origami aggregation and structural damage. For this, both volume fractions were also analyzed by AFM. As can be seen in Figure 2c, the DNA origami remain mostly intact and do not form large aggregates. Furthermore, the bottom 20% fractions on average show a larger surface coverage than the top 80% fractions. However, with the exception of the DNA origami triangles, the overall increase in surface coverage is not dramatic, which is in line with the concentration measurements. 

**Figure 3:**
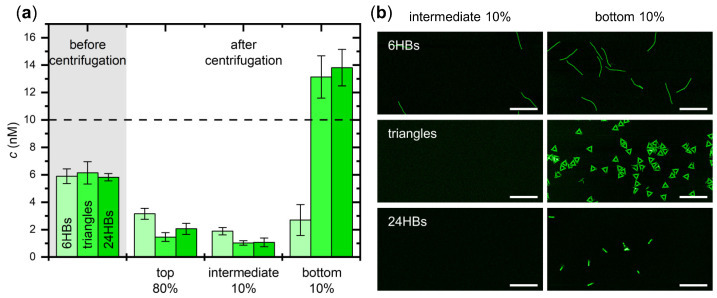
Assessing the formation of DNA origami precipitates in the bottom 20% fraction. (**a**) Concentrations of the DNA origami 6HBs, triangles, and 24HBs before and after centrifugation in 3 M (NH_4_)_2_SO_4_ determined by UV absorption. The horizontal broken line indicates the nominal starting concentration; (**b**) Representative AFM images of the different DNA origami nanostructures in the intermediate 10% and the bottom 10% fractions after centrifugation in 3 M (NH_4_)_2_SO_4_. Scale bars are 500 nm and height scales are 4 nm (6HBs), 3 nm (triangles), and 7 nm (24HBs), respectively.

In order to better assess the formation of precipitates at the bottom of the test tubes, we repeated the experiments described in Figure 2a but split the samples into three fractions, i.e., the top 80%, the bottom 10%, and the intermediate 10%. As can be seen in Figure 3a, the concentrations of the DNA origami triangles and 24HBs determined for the bottom 10% exceed not only the concentrations in the other fractions but also the original concentration of 10 nM. This is particularly noteworthy for the 24HBs, which in the previous experiments (Figure 2b) did not show any enrichment in the lower 20% fraction. The fact that the results in Figure 3a now reveal a strong enrichment only in the bottom 10%, but not in the intermediate 10% fraction, further supports the above interpretation that ammonium sulfate-induced salting-out leads to the formation of solid or semi-solid precipitates at the bottom of the tubes. For the 6HBs, the concentration in the bottom 10% is comparable to the concentrations in the top 80% and the intermediate 10%, which may indicate that the corresponding precipitates are even less soluble. The corresponding AFM images shown in Figure 3b are qualitatively in line with the concentration measurements. In all three cases, the bottom 10% fractions show comparatively large numbers of mostly intact DNA origami nanostructures. In the intermediate 10% fraction, however, no DNA origami are observed at all for the triangles and 24HBs, and for the 6HBs, the number of visible DNA origami is markedly reduced compared to the bottom 10% fraction. In sum, these experiments suggest that centrifugation in 3 M (NH_4_)_2_SO_4_ results not only in the concentration the DNA origami nanostructures at the bottom of the tube but also in the formation of solid or semi-solid precipitates.

**Figure 4:**
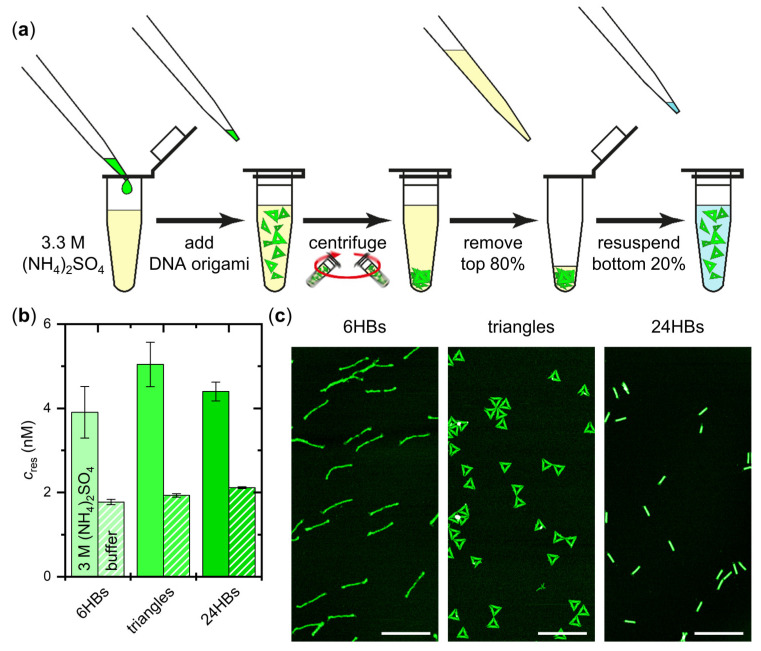
Resuspension of the concentrated DNA origami nanostructures in (NH_4_)_2_SO_4_-free buffer. (**a**) Workflow of DNA origami concentration and resuspension. After centrifugation in 3 M (NH_4_)_2_SO_4_, the top 80% of the sample are discarded and (NH_4_)_2_SO_4_-free assembly buffer is added to the bottom 20% to restore the total sample volume of 100%. The resuspended sample is then further analyzed using UV absorption and AFM; (**b**) Concentrations of the DNA origami 6HBs, triangles, and 24HBs after concentration and resuspension determined by UV absorption. Concentrations obtained after centrifugation in the presence of 3 M (NH_4_)_2_SO_4_ (solid fill) and in assembly buffer without additional salt (pattern fill) are compared; (**c**) Representative AFM images of the different DNA origami nanostructures after concentration in 3 M (NH_4_)_2_SO_4_ and resuspension. Scale bars are 500 nm and height scales are 4 nm (6HBs), 3 nm (triangles), and 7 nm (24HBs), respectively.

Therefore, we next sought to test whether intact DNA origami nanostructures can be recovered from those solid or semi-solid precipitates. To this end, we discarded the top 80% fraction after centrifugation and kept only the bottom 20%. Then, (NH_4_)_2_SO_4_-free assembly buffer was added to restore the original 100% sample volume (see Figure 4a). After gentle mixing, the DNA origami concentration in the resuspended sample was determined. For all three DNA origami shapes, concentrations between about 4 and 5 nM were obtained (see Figure 4b). Assuming that all precipitated DNA origami nanostructures could be resuspended this way, these values suggest that 40 to 50% of the DNA origami present in the original sample solution were concentrated in the bottom 20% fractions. In contrast, identically treated samples without any (NH_4_)_2_SO_4_ show lower concentrations around 2 nM (Figure 4b), which is the expected value for a 1:5 dilution of a non-precipitated sample. While these results suggest efficient resuspension of the precipitated DNA origami nanostructures in 3 M (NH_4_)_2_SO_4_, AFM further verifies that the resuspended DNA origami remain structurally mostly intact during precipitation and resuspension and do not form larger aggregates (Figure 4c). These results thus demonstrate the recovery of the precipitated DNA origami nanostructures at high yields and the possibility of using ammonium sulfate-induced salting-out for transferring DNA origami from one buffer to another.

**Figure 5:**
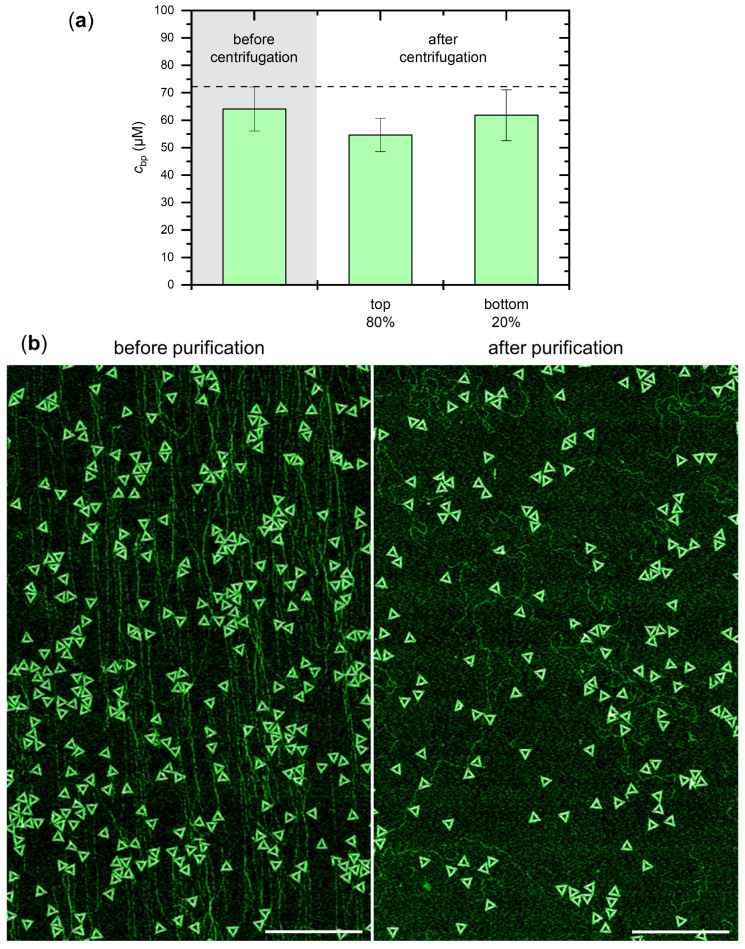
Effect of (NH_4_)_2_SO_4_ on genomic dsDNA. (**a**) Concentrations (in base pairs) of salmon testes DNA before and after centrifugation in 3 M (NH_4_)_2_SO_4_ determined by UV absorption. The horizontal broken line indicates the nominal starting concentration; (**b**) Overview AFM images of a mixture of DNA origami triangles and salmon testes DNA before (left) and after ammonium sulfate purification (right). After centrifuging the mixture in 3 M (NH_4_)_2_SO_4_, the top 95% of the sample volume was discarded and an equivalent amount of (NH_4_)_2_SO_4_-free assembly buffer was added to the remaining 5%. Scale bars are 1 µm and height scales are 1.5 nm.

Next, we tested whether the applied approach can also be used to precipitate genomic dsDNA. For this, we chose salmon testes DNA with a GC content of 41% that is similar to that of the DNA origami nanostructures (see Figure 1d). As can be seen in Figure 5a, centrifugation in 3 M ammonium sulfate did not result in any pronounced precipitation and only small differences in the DNA concentrations of the top 80% and the bottom 20% volume fractions. Motivated by this selectivity for DNA origami nanostructures, we finally tried to purify DNA origami triangles from a complex mixture containing a two-fold excess of genomic dsDNA (in base pairs) over DNA origami nanostructures (5 nM) in 3 M (NH_4_)_2_SO_4_. In the corresponding AFM image of the mixture in Figure 5b (left), one can clearly see the DNA origami triangles as well as several long dsDNA fragments. This mixture was then purified by centrifugation, after which the top 95% fraction of the sample volume was discarded. The remaining bottom 5% fraction was mixed with (NH_4_)_2_SO_4_-free assembly buffer to resuspend the precipitated DNA. The corresponding AFM image of the purified mixture in Figure 5b (right) reveals a notably decreased surface density of dsDNA fragments relative to that of the DNA origami triangles. This is because of the selective precipitation of the DNA origami nanostructures, which thus have a higher concentration in the resuspended sample than the non-precipitated genomic dsDNA. This demonstrates the potential of ammonium sulfate precipitation in separating DNA nanostructures from complex mixtures. Even though there is still a notable amount of dsDNA fragments present in the sample, their concentration can be reduced further by subjecting the sample to multiple precipitation steps.

## 3. Discussion

Salt ions play important roles in stabilizing the native conformations of proteins [56] and nucleic acids [57]. In the case of dsDNA, salt ions are required to facilitate hybridization of the two single strands by screening the electrostatic repulsion between the negatively charged backbone phosphates. Therefore, the DNA melting temperature usually increases with salt concentration [54,57]. However, the presence of salt ions may also modulate the activity of other cosolutes such as chaotropic agents and thereby promote DNA melting [54]. Furthermore, salt ions may also induce structural transitions in polyelectrolytes, which have been studied intensively for the globule (collapsed) to coil (extended) conformation [58], where multivalent ions are particularly effective in driving these transitions. The entropically favored release of counterions from oppositely charged polyelectrolytes was identified as an important driving force in coacervate formation [59]. In contrast to these studies, the rigidity of the DNA origami nanostructures prevents aggregation from coupling to large conformational changes. Furthermore, also multivalent ions and oppositely charged polyelectrolytes are absent in our system. Instead, the salting-out of DNA origami nanostructures resembles the well-known non-denaturing ammonium sulfate precipitation of proteins [46], combining the chaotropic ammonium cation and the kosmotropic sulfate anion, thereby dehydrating hydrophobic surfaces at which aggregation nucleates (for a review see [60]). The likely correlate in the DNA origami nanostructures is the structurally constrained water in the DNA grooves. In association with the ammonium cation, the DNA origami may adopt a charge-neutral ordered hydration shell of low entropy similar to water at hydrophobic protein surfaces. The neutralized DNA origami may thus associate to minimize surface contact with the solvent by forming a shared hydration layer via aggregation, thereby releasing some of the DNA-bound low entropy water similar to protein–protein association and entrapping the ammonium cation. As solvated ions from the bulk would be excluded from this inner hydration layer, an ion-excluded volume potential builds up as an osmotic pressure (that of the salt solution), which will further stabilize the DNA origami aggregates [61]. 

Since we did not observe precipitation of genomic dsDNA under equivalent conditions (see Figure 5a), we assume that this mechanism is specific for the compact and particle-like DNA origami nanostructures that can form intermolecular contacts with large contact areas, whereas dsDNA behaves more like a traditional polyelectrolyte. In this context, however, the different molecular weights of the genomic dsDNA fragments and the DNA origami nanostructures may play a role as well. The salmon testes DNA used in this study is composed of sheared fragments with various sizes up to several thousand base pairs. The AFM image in Figure S2 reveals that the majority of fragments in our sample are comparatively long with lengths of several microns. Therefore, these long fragments have molecular weights similar to those of the DNA origami nanostructures. Nevertheless, precipitation of salmon testes DNA is almost absent in our experiments (see Figure 5a), which indicates that ammonium sulfate precipitation of DNA origami nanostructures is facilitated not predominantly by their large molecular weight but rather by their compact structure. 

While we could demonstrate the separation of DNA origami nanostructures from genomic dsDNA in Figure 5b, it remains to be seen whether ammonium sulfate precipitation may also be employed to separate different DNA nanostructures based on their molecular weights. In this context, additional design and structure-specific factors may have to be taken into account as well. For instance, since the kosmotropic ions interact with DNA-bound water molecules, differences in the hydration shells of different DNA nanostructures may also play an important role. The hydration shell of dsDNA is known to be highly dynamic and heterogeneous [62]. Spatial heterogeneity is caused by the effects of the different hydrophilic and hydrophobic groups in the backbone and the major and minor grooves. Temporal heterogeneity, on the other hand, results from fluctuations in duplex conformation and in particular the groove widths [62]. The parallel arrangement of DNA duplexes in a DNA origami, which are locked in place by periodic backbone crossovers, will undoubtedly affect these fluctuations, and therefore the structure and dynamics of the hydration shell. Furthermore, local differences in the sequences, conformation, and mechanical properties of the different duplexes will result in lateral variations in the hydration shell of the DNA origami surface over nanometer length scales [11]. The overall shape and superstructure of the DNA origami may thus have some effect on their response to kosmotropic salts as well. Pronounced superstructure-dependent effects have for example been observed in the interaction of DNA origami nanostructures with different cations [52,63] and various DNA-binding molecules, in particular groove binders [64], peptides [65], and enzymes [66,67,68]. In the latter examples, the local and global mechanical properties of the DNA origami nanostructures were found to have a strong effect on the interaction, as those directly affect conformational flexibility and groove accessibility [64,66]. In the current experiments, however, such superstructure-dependent effects appear to be only of minor importance. Nevertheless, the data presented in Figure 2, Figure 3 and Figure 4 collectively suggest that on average the DNA origami 6HBs are slightly less affected by the presence of ammonium sulfate, so that somewhat higher concentrations in the top 80% fractions (Figure 2b and Figure 3a) and marginally lower concentrations after resuspension (Figure 4b) are obtained. The quasi-1D 6HBs are closer to linear dsDNA and exhibit a higher flexibility than the more rigid 2D triangles and the 3D 24HBs. Furthermore, while planar triangles, for instance, can form larger inter-molecular contact areas (entailing a larger excluded volume), the lower shape complementarity of the 6HBs will hinter their close packing against each other. It is therefore not surprising that their behavior in the presence of ammonium sulfate is closer to that of genomic dsDNA, even though the differences are rather small.

The purpose of the current study was to establish whether DNA origami nanostructures are susceptible to ammonium sulfate-induced salting-out. While the presented results clearly show salting-out taking place, the employed protocols have not been optimized in any way. The results shown in Figure 4b indicate that after centrifugation for 90 min at 14,000 rcf, at least 40 to 50% of the total DNA origami amount is concentrated in the bottom 20% of the sample volume. Different centrifugation conditions, however, may result in stronger precipitation, so that higher DNA origami concentrations may be obtained. The same holds also for the resuspension of the concentrated DNA origami nanostructures. In the present work, the samples were gently mixed after addition of (NH_4_)_2_SO_4_-free buffer and the resulting DNA origami concentrations were measured immediately. Therefore, we cannot be certain that solid or semi-solid precipitates at the bottom of the sample tube were completely dissolved. It remains to be seen whether higher recovery yields can be obtained by giving the precipitates more time to dissolve.

In the experiments of this work, we have employed the highest ammonium sulfate concentration we could achieve. It is not clear, however, whether such a high concentration is indeed necessary for precipitating DNA origami nanostructures with molecular weights around 5 MDa. Further experiments with different, well-defined DNA nanostructures are required in order to establish a correlation between molecular weight and the ammonium sulfate concentration required for precipitation. This fundamental knowledge may then enable the separation of differently sized DNA nanostructures from complex mixtures, similar to the case of protein extraction from cell lysates.

A general issue in ammonium sulfate precipitation is that even after transfer into (NH_4_)_2_SO_4_-free buffers, the resulting solution will still exhibit a significant amount of residual (NH_4_)_2_SO_4_. In the resuspension experiments described in Figure 4, the original (NH_4_)_2_SO_4_-containing buffer was diluted 1:5 with (NH_4_)_2_SO_4_-free buffer, resulting in a final (NH_4_)_2_SO_4_ concentration in the resuspended sample of 0.6 M. This residual (NH_4_)_2_SO_4_ concentration could in general be further reduced by using lower starting concentrations. However, because the (NH_4_)_2_SO_4_ concentration required for salting-out a given biomolecule scales with its molecular weight (see above), we doubt that the (NH_4_)_2_SO_4_ concentration can be reduced substantially. In protein precipitation, residual (NH_4_)_2_SO_4_ is typically removed afterwards by dialysis [46]. In the case of DNA origami precipitation, applying such an additional purification step will only make sense if the aim of the precipitation step is to separate different species with different molecular weights and not just their purification from excess staples. When it comes to such routine purification and concentration purposes, ammonium sulfate-induced DNA origami precipitation will be most useful for applications that can tolerate the residual (NH_4_)_2_SO_4_.

## 4. Materials and Methods

### 4.1. DNA Origami Synthesis and Purification

The assembly of DNA origami triangles [1] and 6HBs [55] was based on a previously published protocol [65]. For this purpose, the 7249 nt M13mp18 scaffold (Tilibit GmbH, München, Germany) and about 200 staple strands (Eurofins Genomics GmbH, Ebersberg, Germany) were mixed with a molar ratio of 1:10 in 10 mM Tris buffer (Sigma-Aldrich Chemie GmbH, Steinheim, Germany) containing 10 mM MgAc_2_ (Sigma-Aldrich Chemie GmbH, Steinheim, Germany). The pH of the Tris buffer was adjusted to 8.0 with acetic acid (Merck KGaA, Darmstadt, Germany). The one-pot assembly reaction was performed by gradually decreasing the temperature starting from 80 °C to room temperature over 90 min using a Primus 25 advanced thermocycler (PEQLAB Biotechnologie GmbH, Erlangen, Germany).

The assembly of 24HBs was based on a previously published protocol [33]. Here, the 7560 nt M13mp18 scaffold (Tilibit GmbH, München, Germany) and about 200 staple strands (Eurofins Genomics GmbH, Ebersberg, Germany) were mixed with a molar ratio of 1:10 in 10 mM Tris buffer containing 10 mM MgAc_2_. The pH of the Tris buffer was adjusted to 8.0 with acetic acid. The one-pot assembly reaction was performed by gradually decreasing the temperature starting from 65 °C to 59 °C, over 90 min, followed by a lower gradient from 59 °C to 40 °C, over 60 h, using a Primus 25 advanced thermocycler.

The same purification protocol was applied for all the three DNA origami nanostructures. To this end, the unpurified DNA origami samples were filtered by using Amicon Ultra-0.5 mL spin filters with 100 kDa molecular weight cut-off (Merck KGaA, Darmstadt, Germany). The concentrations of the purified DNA origami were determined as described in Section 4.3 and adjusted to a concentration of 100 nM for each DNA origami nanostructure.

### 4.2. Salting-Out and Resuspension Experiments

100 µL samples were prepared by mixing 75 µL of 4 M (NH_4_)_2_SO_4_ solution (Merck KGaA, Darmstadt, Germany) or 4 M NaCl (VWR International S.A.S., Fontenay-sous-Bois, France), 9 µL of 100 mM Tris buffer containing 100 mM MgAc_2_, 6 µL HPLC-grade water (VWR International S.A.S., Fontenay-sous-Bois, France), and 10 µL of the purified DNA origami sample (100 nM). For the (NH_4_)_2_SO_4_-free controls, the 4 M (NH_4_)_2_SO_4_ solution was replaced by HPCL-grade water. The solutions were centrifuged by using a VWR microcentrifuge at 14,000× *g* rcf for 90 min at 18 °C. Next, the solutions were carefully split into two fractions consisting of the top 80% and the bottom 20% of the total sample volume, respectively. Both fractions were gently mixed with a pipette five times in order to disperse segregated DNA origami. 

In an additional set of experiments (Figure 3), the samples were split into three fractions after centrifugation, i.e., the top 80%, the bottom 10% and the intermediate 10% of the total sample volume. To dissolve the solid or semi-solid DNA origami precipitates, the 10% fractions were thoroughly vortexed at 2800 rpm. 

For the resuspension experiments in Figure 4, the 20% bottom fractions were mixed with 10 mM Tris buffer containing 10 mM MgAc_2_ to yield the original sample volume of 100%. For the experiments with genomic dsDNA in Figure 5, DNA from salmon testes was used (Thermo Fisher GmbH, Kandel, Germany).

### 4.3. Concentration Measurements

DNA concentrations were measured using an Implen Nanophotometer P330 (Implen GmbH, München, Germany) operated in dsDNA mode. For each sample, a 1 µL aliquot was analyzed using the P-Class Submicroliter Cell P330 (Implen GmbH, München, Germany) with corresponding DNA-free buffer as the blank. The DNA concentrations were determined from the DNA absorption peak at 260 nm after subtraction of the background absorption at 320 nm. Example UV-vis spectra are shown in Figure S1.

### 4.4. AFM Imaging

A 1 µL aliquot of each fraction was deposited onto freshly cleaved mica and immediately covered with 100 µL of 10 mM Tris buffer containing 10 mM MgAc_2_. After 2 min of incubation, the samples were rinsed with about 6 mL of HPLC-grade water and dried under a stream of Ar. For the separation experiments in Figure 5b, 2 µL aliquots were used and incubated for 5 min to account for the lower DNA origami concentration. AFM imaging was performed in air using a Bruker Dimension ICON (Bruker France S.A.S., Wissembourg, France) in ScanAsyst Peak-Force Tapping mode with ScanAsyst-Air cantilevers (Bruker AFM Probes, Camarillo, CA, USA). The obtained AFM images were flattened and height-adjusted using Gwyddion 2.52 open-source software [69].

### 4.5. Quantification and Statistical Analysis

The ammonium sulfate experiments were performed in triplicate and the control experiments in duplicate. The results of the concentration measurements are presented as mean values with standard deviations as error bars. Mean values and standard deviations were computed using OriginPro 2020 (OriginLab Corporation, Northampton, MA, USA). 

## Data Availability

The data presented in this study are available on request from the corresponding author.

## Supplementary Materials

The following supporting information can be downloaded at: https://www.mdpi.com/article/10.3390/ijms23052817/s1.
