# Supplementary material for: Salting-Out of DNA Origami Nanostructures by Ammonium Sulfate
Source: Int J Mol Sci. 2022 Mar 4;23(5):2817. doi: 10.3390/ijms23052817 (PMC8911265; doi:10.3390/ijms23052817)
Supplement: Supplementary file 1 [file ijms-23-02817-s001.zip › ijms-1605161-supplementary.pdf]

Supplementary Materials

Salting-out of DNA Origami Nanostructures by Ammonium Sulfate

Marcel Hanke [1], Niklas Hansen [1,†], Ruiping Chen [1], Guido Grundmeier [1], Karim Fahmy [2,3] and Adrian Keller [1,]*

[1] Paderborn University, Technical and Macromolecular Chemistry, Warburger Str. 100, 33098 Paderborn, Germany

[2] Helmholtz-Zentrum Dresden-Rossendorf, Institute of Resource Ecology, Bautzner Landstrasse 400, 01328 Dresden, Germany

[3] Technische Universität Dresden, Center for Molecular and Cellular Bioengineering, 01062 Dresden, Germany

[†] Present address: Department of Biophysical Chemistry, J. Heyrovsky Institute of Physical Chemistry, Czech Academy of Sciences, Dolejškova 3, 182 23 Prague 8, Czech Republic

* Correspondence: adrian.keller@uni-paderborn.de, Tel.: +49-5251-605722

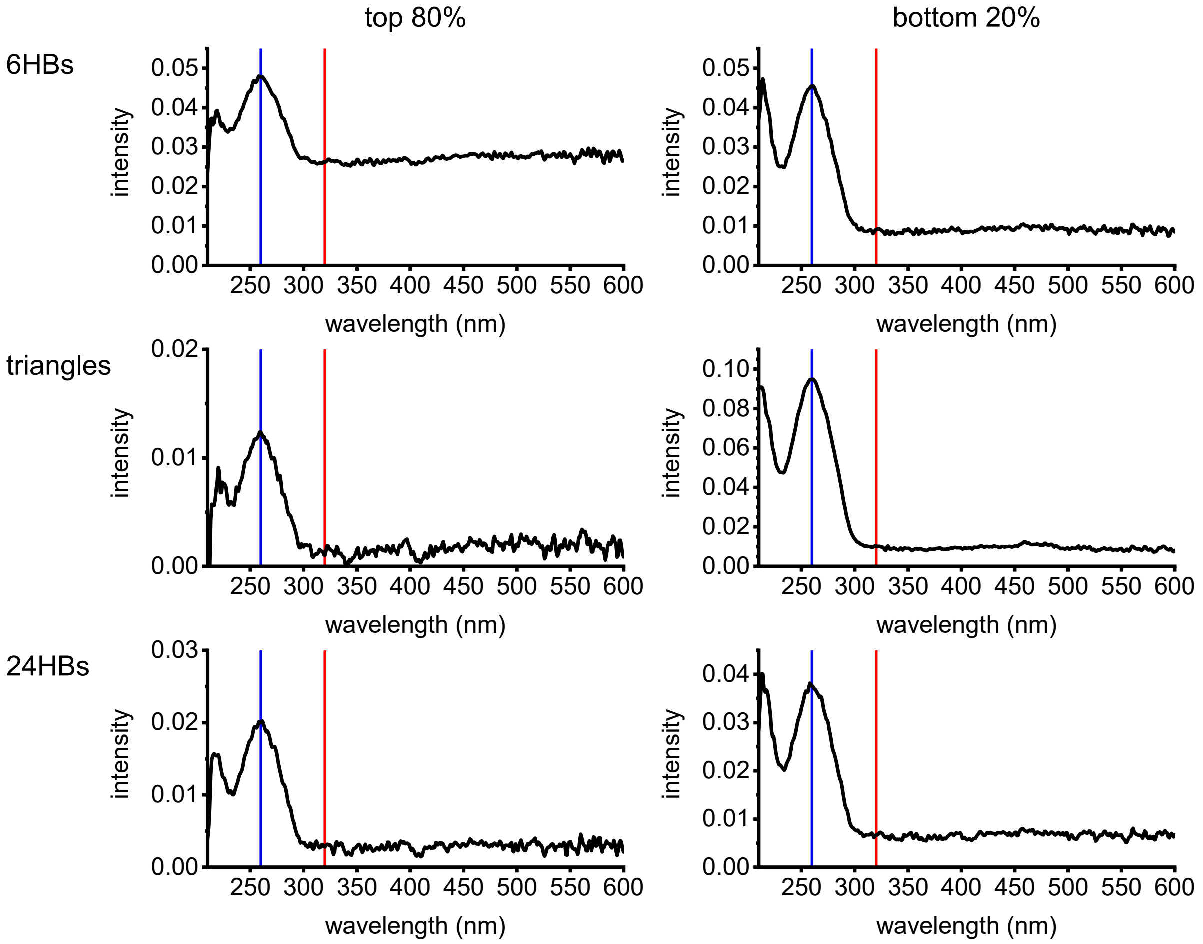

Figure S1: *Example UV-vis spectra of DNA origami 6HBs (top), triangles (center), and 24HBs (bottom) after centrifugation in 3 M ammonium sulfate. The spectra on the left and right were recorded in the top 80% and bottom 20% fraction of the total sample volume, respectively. The vertical lines indicate the wavelengths used for DNA quantification (260 nm, blue) and background subtraction (320 nm, red), respectively.*

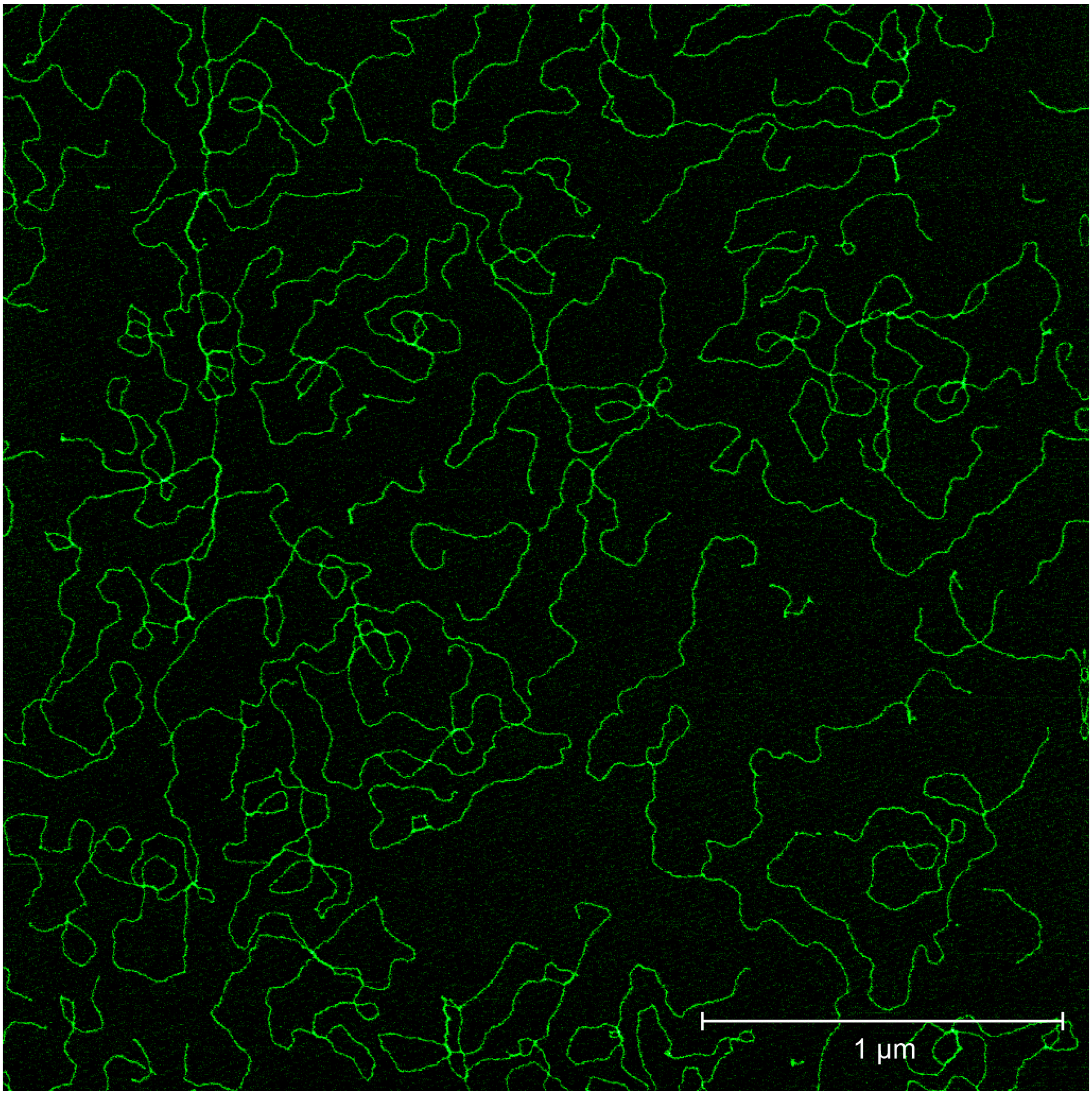

Figure S2: *Representative AFM image of genomic dsDNA from salmon testes.*
